# Telomere length and telomere repeat-binding protein in children with sickle cell disease

**DOI:** 10.1038/s41390-021-01495-6

**Published:** 2021-04-06

**Authors:** Mohamed E. Suliman, Mohammed G. A. Ansari, Mohamed A. Rayis, Muaawia A. Hamza, Abdullah A. Saeed, Abdul Khader Mohammed, Nasser M. Al-Daghri

**Affiliations:** 1 Faculty of Medicine, King Fahad Medical City, Ministry of Health, Riyadh, Kingdom of Saudi Arabia; 2 Chair for Biomarkers of Chronic Diseases, Biochemistry Department, College of Science, King Saud University, Riyadh, Kingdom of Saudi Arabia; 3 Children Hospital, King Fahad Medical City, Ministry of Health, Riyadh, Kingdom of Saudi Arabia; 4 Research Center, King Fahad Medical City, Ministry of Health, Riyadh, Kingdom of Saudi Arabia; 5 Sharjah Institute for Medical Research, University of Sharjah, Sharjah, United Arab Emirates

## Abstract

**Background:**

This study aimed to assess the telomere length and plasma telomere repeat-binding factor 2 (TRF2) levels in addition to other inflammatory markers in children with sickle cell disease (SCD).

**Methods:**

We enrolled 106 children (90 SCD and 26 controls) aged 1–15 years from the Hematology unit of King Fahad Medical City (KFMC), Saudi Arabia. Genomic DNA extracted from blood and leukocyte TL was determined using quantitative reverse transcription PCR, whereas TRF2, C-reactive protein, interleukin-6, and DNA oxidative damage were determined by using respective commercially available assays.

**Results:**

Leukocyte TL was inversely correlated with age in the SCD patients (*r* = −0.24, *P* = 0.02) and the controls (*r* = −0.68, *P* < 0.0001). In addition, SCD patients had significantly shorter TL (7.74 ± 0.81 kb) (*P* = 0.003) than controls (8.28 ± 0.73 kb). In contrast, no significant difference in TL among the SCD genotypes (HbSS and HbSβ0) has been observed. A modest, positive correlation was seen between TL and reticulocyte % (*r* = 0.21; *P* = 0.06). There were no significant differences in the TL and TRF2 concentrations between subjects with HbSS and HbSβ^0^ genotypes.

**Conclusions:**

Short leukocyte TL was significantly associated with SCD. An inverse association was observed between TL and hemoglobin. Hydroxyurea treatment revealed no impact on TL.

**Impact:**

- This study explored the TL and plasma TRF2 in Saudi children with SCD. This is the first documentation that SCD children have shorter TL than their healthy counterparts, and no association between TL and TRF2 has been observed.
- Hydroxyurea treatment showed no impact on TL in children with SCD.
- This study is the first of its kind in children with SCD.
- It will pave the way for another study with a larger sample size in a diverse population to scrutinize these findings better.

## Introduction

Telomeres are noncoding nucleoprotein structures that cover the ends of repetitive nucleotide (TTAGGG) sequences and stabilize the genome^1,2^ by capping chromosomal ends and protecting them from fusion and degradation. This mechanism prevents the loss of genomic information during chromosome replication.^3^ Telomeres can span from 4 to 10 kb in human cells, depending on the chromosome cell type and genetic variation.^4^ Each cell division leads to a telomere shortening (5–100 bp),^5,6^ with the average rate of decline being more significant in men than in women.^7^ This shortening continues until the telomere reaches a critical length,^8^ which in turn triggers cell-cycle arrest, leading to senescence or apoptotic cell death. Such a unique feature enables telomeres to serve as a mitotic clock by limiting cells’ ability to divide.

Shelterin is a six-subunit protein complex, which shapes and safeguards the function of telomeres. It acts in accordance with several other associated factors to facilitate the protection, replication, and processing of chromosome end after DNA replication.^9^ At the chromosome ends, shelterin prevents DNA damage response (DDR) activation and regulates telomerase activity.^10^ This complex interacts with components of the DDR pathway in addition to DDR proteins (telomere repeat-binding factors (TRF) 1 and 2), which bind directly to the TTAGGG repeat DNA. Shelterin is anchored to the chromosomal end by the double-strand DNA-binding proteins TRF1 and TRF2. Both TRFs are considered as negative regulators of the telomeric length (TL),^11,12^ thereby inhibiting or restricting telomere elongation.^13^

In addition, TRF2 has unique functions in the telomere as a stabilizer of the protruding G-string, and it can prevent telomeric fusions.^7^ The progressive shortening of TL is the product of TRF2 overexpression, similar to the phenotype observed with TRF1.^12,14^ This may cause premature senescence^7,14^ and activate the apoptotic cascade mediated by the protein kinase ATM and p53.^15^

Sickle cell disease (SCD), a hereditary blood disorder, poses considerable health and socioeconomic burden and may lead to various acute and chronic complications.^16,17^ SCD like other vascular diseases is characterized by endothelial dysfunction, of which telomerase might play a significant role.^18,19^ Endothelial senescence is modulated by telomerase activity, and the pro-oxidant environment in SCD might lead to telomere shortening. Moreover, serum markers showed evidence of chronic inflammation status in SCD.^20^ It is known that inflammation promotes endothelial adherence to sickle erythrocytes. Increased systemic inflammation consistent with vascular injury is associated with decreased leukocyte TL.^21,22^ Given the ongoing inflammation and oxidative stress in the pathophysiology of SCD,^20^ it is plausible that age-adjusted TLs could be a marker of SCD severity. On the contrary, an in vitro study showed that hydroxyurea (HU), an important drug therapy for sickle cell anemia that prolongs survival, affects telomere replication and reduces the telomere DNA synthesis rate through a mechanism that may involve TRF2 direct modification.^23^

So far, two studies determined leukocyte TL in adult SCD patients^24,25^ and showed conflicting results. Interestingly, no study has been reported in children with SCD. To fill this gap, the rationale of the current study aimed to evaluate the TL and plasma TRF2 in children with SCD and assess the effects of HU therapy on TL and plasma TRF2 concentration. We also determine the changes in TL and plasma TRF2 in relation to their hematological parameters, systemic inflammation, and oxidative DNA damage.

## Methods

### Study population

The study population includes 106 children (90 SCD patients and 26 healthy controls) aged 1–15 years old, recruited from the Hematology Unit, Children Hospital, King Fahad Medical City (KFMC), Riyadh, Kingdom of Saudi Arabia (KSA). The KFMC Institutional Review Board (IRB) approved this study (IRB approval #14-223). Written informed consent from parents and assent from children were obtained prior to inclusion in the study. All methods were performed in accordance with the relevant guidelines and regulations.

Venous blood samples were collected and transferred in appropriate tubes and concomitant with the clinical evaluation data collection. Peripheral blood for DNA extraction was collected in an EDTA tube, which was immediately transferred to the biobank of Chair for Biomarkers of Chronic Diseases, King Saud University, Riyadh, KSA, whereas blood for plasma/serum was obtained in plain tubes. Separated plasma was aliquoted and stored at –80 °C until further analysis.

The hematological parameters were determined by routine methods at KFMC laboratories. TRF2 concentrations were quantified using the enzyme-linked immunosorbent assay (ELISA) Kit (LifeSpan Bioscience Inc., Seattle, WA). Plasma C-reactive protein (CRP) and interleukin-6 (IL-6) were determined as inflammation markers using available ELISA Kits (R&D Systems Inc., MN). DNA oxidative damage was determined by measuring serum 8-hydroxy-2′-deoxyguanosine (8-OHdG) using an ELISA Kit (Cell Biolab Inc., San Diego, CA).

### Telomere measurement

Genomic DNA was isolated from the frozen whole blood using DNeasy Blood and Tissue Kit (Qiagen Hilden, Germany). The Nanodrop spectrophotometer was used to determine DNA concentration and purity (260/280). TL determination was done as described in the previous studies of Al-Attas and colleagues done on Arab populations.^26–28^ TL was assessed using a real-time PCR Bio-Rad CFX96 Real-Time PCR Detection System (Bio-Rad, Milan Italy) as previously described.^29^ In brief, the assay involved comparing the abundance of telomere DNA to an internal reference gene of invariant copy number for each sample and by further comparison of normalized values between DNAs of different sources. Serial dilutions of two reference DNA samples (MRC5 and KE27) were used to construct standard curves of amplification using *GAPDH* (fixed copy number reference gene) and telomere primer pairs. All the reactions were performed in 96-well plates in a final reaction volume of 20 μl, containing 2X ready-mix (KAPA Biosystems) containing SYBR green and 5 pmol of each primer. Each DNA sample was analyzed in triplicates alongside two reference DNA samples and non-template control.

The PCR cycles were as follows: 3 min at 95 °C, followed by 40 cycles of 15 s at 95 °C, 30 s at 56 °C, and 30 s at 72 °C. The data were obtained as the cycle threshold (Ct) values. The efficiency of amplification values derived from the standard curves was used to calculate the differences in the abundance of the telomere amplicons for each amplicon. These were corrected by calibration against the known TL in the reference DNAs.

### Statistical analysis

The data were analyzed using SPSS for windows version 11.5 (SPSS Inc., Chicago, IL). All values are expressed as mean ± standard deviation (SD), unless otherwise indicated. A *P* value <0.05 was considered to be statistically significant. Comparisons between two groups were assessed for continuous variables with the Student’s unpaired *t* test, Mann–Whitney test, or *χ*^2^ test, as appropriate. Spearman’s rank correlation or Pearson’s correlation was used as appropriate to determine correlations between the variables.

## Results

The current study was conducted on 90 SCD (54% were males, with a mean age of 8 years) and 26 healthy children (48% males, with a mean age of 9 years). Based on the genotype, the SCD group was further divided into two groups—HbSS (78) and HbSβ^0^ (12) patients, as shown in Table 1. Thirty-nine patients (43%) were on HU with a median dose of 500 g/day (usual dose range: 20–25 mg/kg/day and not exceeding 35 mg/kg/day) and 52% of the patients were classified as having severe SCD. Classification of disease severity criteria was adopted from Quinn et al.,^30^ which is based on hemoglobin abnormalities and named as sickle cell anemia (HbSS), sickle β-zero thalassemia (HbSβ0), sickle hemoglobin-C disease (HbSC), and sickle β-plus thalassemia (HbSβ+).

**Table 1:** Clinical characteristics, hematological, inflammation, and oxidative stress parameters in children with sickle cell disease.

*Parameters*	All SCD Subjects	HbSS	HbSβ^0^	*P* value
N	90	78	12	
Male:female	49/41	45/33	4/8	0.10
Age (years)	8.4 ± 3.5	8.5 ± 3.6	8.0 ± 3.2	0.51
Hydroxyurea (yes)	39 (43%)	36 (46%)	3 (25%)	0.14
Hemoglobin (g/dL)	8.5 ± 1.1	8.5 ± 1.1	8.5 ± 0.9	0.90
Reticulocytes (%)	11.4 ± 5.9	12.0 ± 6.0	7.4 ± 4.2	0.003
Platelets (10^9^/L)	466 ± 179	459 ± 180	515 ± 171	0.35
White blood cells (10^9^/L)	12.4 ± 4.5	12.5 ± 4.7	11.4 ± 2.9	0.47
Lactate dehydrogenase (mmol/L)	544 ± 144	553 ± 141	478 ± 157	0.14
Ferritin (ng/mL)	535 ± 866	567 ± 914	289 ± 211	0.36
25(OH)D (nmol/mL)	32 ± 18	31 ± 18	36 ± 18	0.39
Telomere length (kb)	7.74 ± 0.81	7.74 ± 0.81	7.76 ± 0.85	0.94
TRF2 (ng/mL)	6.37 ± 2.33	6.36 ± 2.35	6.42 ± 2.27	0.51
IL-6 (pg/mL)	5.76 ± 5.53	6.13 ± 5.78	3.13 ± 1.85	0.15
CRP (mg/L)	4.88 ± 3.86	5.11 ± 3.88	3.30 ± 3.54	0.18
8-OHdG (ng/mL)	5.14 ± 3.52	5.02 ± 3.67	5.89 ± 2.26	0.60

*TRF2* telomeric repeat-binding factor 2, *IL-6* interleukin-6, *CPR* C-reactive protein, *8-OHdG* 8-hydroxy-2′-deoxyguanosine.

**Fig. 1:**
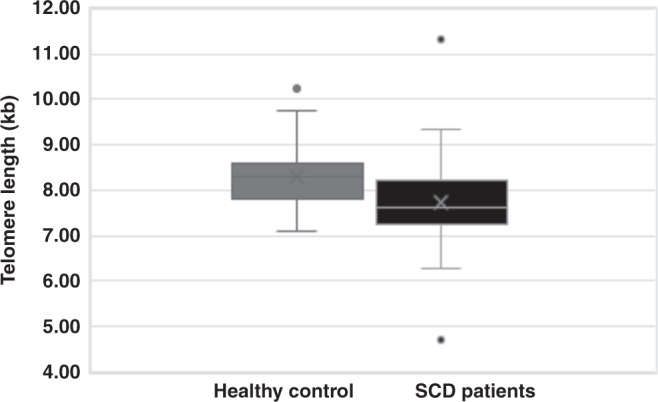
Leukocyte TL in SCD (grey) versus Control (black).

Our results showed that leukocyte TL was inversely correlated with age in the SCD patients (*r* = −0.24, *P* = 0.02) and the controls (*r* = −0.68, *P* < 0.0001). However, telomere length did not significantly differ between males and females in the SCD patients and the control group. The leukocyte TL was significantly shorter (*P* = 0.003) in the SCD patients (7.74 ± 0.81 kb) than their healthy counterparts (8.28 ± 0.73 kb) (Fig. 1). No significant difference in age and sex between the SCD patients and controls were found. In addition, no significant association was found between TL and TRF2.

Based on the phenotypes, patients were divided into two groups, as in Table 1. The results show no significant differences in the TL and TRF2 concentration between the patients with HbSS and those with HbSβ^0^. The patients with HbSS showed a higher percentage of reticulocytes than the patients with HbSβ^0^. Nevertheless, there were no significant differences between the two patient groups regarding other hematological parameters, inflammation markers, and 8-OHdG concentrations.

**Fig. 2:**
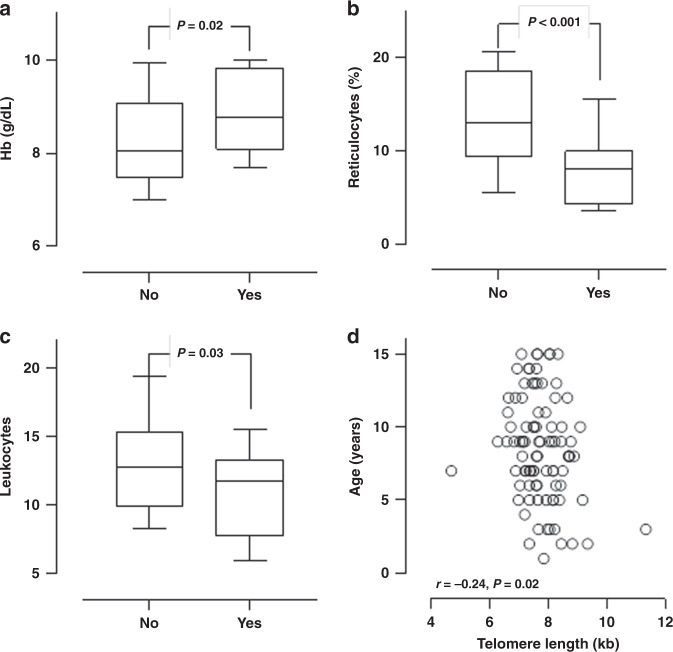
Differences in levels of **a** hemoglobin; **b** reticulocyte and **c** leukocyte among those who received hydroxyurea (yes) and those who did not (no). **d** Association between TL and age in SCD children.

Furthermore, the SCD patients were divided into two groups based on HU treatment, as shown in Table 2. The findings showed that there were no significant differences in the TL and TRF2 between both groups. The patients on HU treatment (*n* = 39) were older, had higher Hb, lower reticulocytes (%), and lower white blood cell (WBC) count (Fig. 2). The platelet count, circulating lactate dehydrogenase, ferritin, CRP, IL-6, and 8-OHdG were not significantly different between groups.

**Table 2:** Differences between SCD subjects with or without HU treatment.

*Parameters*	HU	Non-HU	*P* value
N	39	51	
Male:female (%)	21(54%)/18(46)	28(55)/23(5)	0.54
Age (years)	9.8 ± 3.1	7.1 ± 3.6	0.001
Hemoglobin (g/dL)	8.8 ± 1.0	8.3 ± 1.1	0.03
Reticulocytes (%)	8.4 ± 4.6	13.6 ± 6.0	0.001
Platelets (10^9^/L)	469 ± 204	463 ± 160	0.89
White blood cells (10^9^/L)	11.0 ± 3.7	13.4 ± 4.9	0.02
Lactate dehydrogenase (mmol/L)	513 ± 157	568 ± 130	0.10
Ferritin (ng/mL)	406 ± 302	630 ± 1107	0.26
25(OH) D (nmol/mL)	30 ± 17	33 ± 19	0.41
Telomere length (kb)	7.65 ± 0.65	7.82 ± 0.92	0.33
TRF2 (ng/mL)	6.37 ± 2.39	6.33 ± 2.31	0.93
IL-6 (pg/mL)	5.01 ± 5.01	4.87 ± 4.11	0.14
CRP (mg/L)	6.41 ± 4.95	5.82 ± 5.01	0.57
8-OHdG (ng/mL)	4.91 ± 2.87	5.16 ± 3.44	0.73

*HU* hydroxyurea, *TRF2* telomeric repeat-binding factor 2, *IL-6* interleukin-6, *CPR* C-reactive protein, *8-OHdG* 8-hydroxy-2′-deoxyguanosine.

Table 3 shows the associations of TL with hematologic (hemoglobin, reticulocyte counts (RETICS%), and lactate dehydrogenase), inflammation (IL-8, CRP, ferritin, and total WBC counts) and 8-OHdG markers. TL was inversely correlated with Hb and positively correlated with WBC count. A modest, positive association was seen between TL and reticulocyte % (*r* = 0.21; *P* = 0.06). TL did not show a correlation with the plasma levels of other hematological markers, inflammatory markers, and 8-OHdG.

**Table 3:** Correlations of TL in SCD patients.

Parameters	Correlation coefficient	*P* value
Age (years)	−0.24	0.02
Hemoglobin (g/dL)	−0.28	0.01
Reticulocytes (%)	0.21	0.06
Platelets (10^9^/L)	0.18	0.11
White blood cells (10^9^/L)	0.24	0.03
Lactate dehydrogenase (mmol/L)	0.12	0.30
Ferritin (ng/mL)	0.07	0.54
TRF2 (ng/mL)	0.03	0.75
IL-6 (pg/mL)	0.10	0.33
CRP (mg/L)	−0.11	0.33
8-OHdG (ng/mL)	0.11	0.28

*TRF2* telomeric repeat-binding factor 2, *IL-6* interleukin-6, *CPR* C-reactive protein, *8-OHdG* 8-hydroxy-2′-deoxyguanosine.

## Discussion

To the best of our knowledge, this is the first documentation that children with SCD have shorter peripheral blood leukocyte TL compared with age-matched healthy children. Moreover, TL was inversely associated with age in patients with SCD. However, the treatment with HU did not affect TL in children with SCD.

Shortening TL may affect the general health of individuals and is associated with certain disorders. Shortened TL has been found in patients with inherited conditions associated with premature aging^31^ and acquired age-related disorders, including atherosclerotic cardiovascular disease,^32^ diabetes mellitus (DM),^33^ and hematologic malignancies and hematopoietic stem cells.^34^ So far, no consistent finding was reported on peripheral blood leukocyte TL in adult patients with SCD. The findings of the two studies conducted in adult patients were contradicting.^24,25^ Drasar et al.^24^ reported that the TL was longer in adult SCD than in healthy subjects. However, Colella et al.^25^ found that TL in adult patients with SCD was shorter than in healthy adults.

In the current study and contrary to Colella et al.,^25^ we found that children with SCD have shorter TL than healthy subjects. Notably, in our study, the telomere shortening did not differ between subjects with HbSS and HbSβ^0^ genotypes. In contrast, Colella et al.^25^ have reported a significant difference in TL among SCD genotypes, which was shorter in HbSS subjects than in the HbSC and HbSβ subjects. Moreover, Drasar et al.^24^ found adult SCD subjects had longer TL than the HbSC adult subjects.

HU is an orally administered chemotherapeutic agent commonly used for hematological disorders, including SCD. The indications for HU included regular painful episodes (6 or more per years), the history of acute chest syndrome, and severe vaso-occlusive events along with unremitting chronic pain, which cannot be controlled with conservative measures such as severe symptomatic anemia, and a history of stroke/high risk for stroke.

In vitro studies^23^ showed that chronic treatment with HU induces telomere dysfunction through a mechanism that correlates with the post-translational modification of TRF2 and the loss of TRF2 DNA-binding activity, which eventually leads to shortening TL. Contrary to previous findings in adult SCD patients,^24,25^ our study showed short TL in children with SCD, and did not find a difference in leukocyte TL between the patient treated with HU and the untreated group. It may be worth mentioning that our subjects were on treatment for an average of 3 years with a mean dose of 350 mg/day. In adult SCD studies,^24,25^ no treatment duration data have been found; however, a mean dose of 1 g was mentioned in another study.^25^ Therefore, we cannot exclude the treatment duration and the treatment dose to explain our findings. Thus, future studies will be required to verify whether the TL shortening is influenced by the duration and/or treatment dose.

TRFs are indispensable components of the molecular machinery that regulates telomere function. The telomeric ends are controlled by the binding of TTAGGG repeat-binding factors, TRF1 and TRF2, which serve as negative TL regulators. Given these facts, one should expect a direct association between TL and circulatory TRF2 levels. However, such an association was not identified in our study. Moreover, TRF2 concentration was not correlated with hematological, inflammatory, and oxidative stress markers. It has been reported^23^ that HU treatment induces replication stress and altered the association of TRF1 and TRF2 with telomere. Thus, our findings may reflect the influence of therapy, which may have interfered with TL and may mask a plausible association between TL and TRF2. However, to the best of our knowledge, no reports regarding the relationship between TRF2 and TL in SCD subjects have yet been reported. Therefore, further investigation of such a relation is warranted to confirm or refute our findings.

It has been demonstrated that iron has an influence and participate in telomere maintenance. Depletion of some iron-containing proteins such as DNA polymerases Polα, δ, and ε, Regulator of telomere length 1 (RTEL1), and the small subunit of Ribonucleotide reductase (RNR) results in abnormal TL.^35–37^ Moreover, patients with diseases caused by iron deficiency or iron overload also have short TL.^38,39^

Notably, Kozlitina and Garcia^39^ found, besides the inverse association between TL and hemoglobin, that short telomere lengths were associated with lower red blood cell (RBC) counts, larger mean RBC size, increased RBC distribution width, and lower platelet counts in a large urban US population. Interestingly, our study showed an inverse association between TL and hemoglobin concentration, inconsistent with Colella et al.^25^ findings. Moreover, the relationship between TL and RBC parameters was examined in another study by Asklepios. They found that shorter telomere length was associated with lower RBC counts, larger RBC size, and higher hemoglobin concentrations in the healthy adult populations.^40^ Further studies are needed to explore the relationship between telomere shortening and hematological indices in SCD.

Levels of circulatory inflammation markers may associate with a decrease in TL, but this was not observed in the present study. One study^25^ reported an association between TL and inflammation in SCD. They found that TL was not associated with all studied inflammatory markers, but only with IL-8 levels and lymphocytes. Besides the known cytostatic effect of HU, it also has an anti-inflammatory action in SCD.^41^ Moreover, Lanaro et al.^42^ reported the presence of alterations in the gene expressions and productions of several pro- and anti-inflammatory mediators in SCD and notably in patients on HU therapy. In addition to the mentioned effect of SCD on the gene expressions and production of inflammatory cells, HU can reduce the number of high turnover cells, such as neutrophils, platelets, and reticulocytes, and alteration of the circulating levels of inflammatory markers. Therefore, our results may reflect the influence of SCD per se and the cytostatic and anti-inflammatory effects of HU on hematological cells and inflammation markers. In addition, in this study, we measured the circulating levels of 8-OHdG, which acts as a biomarker to detect systemic oxidative DNA damage associated with oxidative stress. However, no significant associations were seen with 8-OHdG and telomere length as well as with all other laboratory variables, including hematological and inflammation markers. On the other hand, it has been reported that 8-OHdG was a major independent predictor for telomere length in type 2 DM (T2DM),^43^ an independent risk factor for telomere shortening in both T1DM and T2DM.^44^

## Limitations

We acknowledge the limitation of the small sample size. Further investigation using a larger number of participants may provide a clearer and interesting insight into the overall cross-talk between TL and SCD. Second, given the modifying effect of α-thalassemia on SCD phenotype and α-thalassemia gene deletion, a study in SCD subjects should be the focus of future studies as they have high frequencies in Saudi Arabian population. Lastly, we are unsure if the study population is geographically representative of the entire children population with SCD. To address these limitations in future studies, subjects from different regions should be recruited. Nevertheless, this study is the first of its kind in children with SCD.

## Conclusion

The findings of this study show that telomeres are shorter in children with SCD than healthy controls, and HU treatment did not influence TL. An inverse association between TL and hemoglobin was observed. TL was not associated with inflammatory markers and oxidative stress markers.

## References

[1] Blackburn EH, Greider CW, Szostak JW (2006). Telomeres and telomerase: the path from maize, Tetrahymena and yeast to human cancer and aging. Nat. Med..

[2] Cech TR (2004). Beginning to understand the end of the chromosome. Cell.

[3] Blasco MA (2005). Telomeres and human disease: ageing, cancer and beyond. Nat. Rev. Genet..

[4] Riethman H (2008). Human telomere structure and biology. Annu. Rev. Genomics Hum. Genet..

[5] Huffman KE, Levene SD, Tesmer VM, Shay JW, Wright WE (2000). Telomere shortening is proportional to the size of the G-rich telomeric 3’-overhang. J. Biol. Chem..

[6] Zhao Y (2009). Telomere extension occurs at most chromosome ends and is uncoupled from fill-in in human cancer cells. Cell.

[7] Dalgård C (2015). Leukocyte telomere length dynamics in women and men: menopause vs age effects. Int. J. Epidemiol..

[8] Zhao Z, Pan X, Liu L, Liu N (2014). Telomere length maintenance, shortening, and lengthening. J. Cell Physiol..

[9] de Lange T (2005). Shelterin: the protein complex that shapes and safeguards human telomeres. Genes Dev..

[10] Martínez P, Blasco MA (2011). Telomeric and extra-telomeric roles for telomerase and the telomere-binding proteins. Nat. Rev. Cancer.

[11] Bianchi A, de Lange T (1999). Ku binds telomeric DNA in vitro. J. Biol. Chem..

[12] Smogorzewska A (2000). Control of human telomere length by TRF1 and TRF2. Mol. Cell. Biol..

[13] van Steensel B, de Lange T (1997). Control of telomere length by the human telomeric protein TRF1. Nature.

[14] van Steensel B, Smogorzewska A, de Lange T (1998). TRF2 protects human telomeres from end-to-end fusions. Cell.

[15] Karlseder J, Broccoli D, Dai Y, Hardy S, de Lange T (1999). p53- and ATM-dependent apoptosis induced by telomeres lacking TRF2. Science.

[16] Alenzi FQ (2020). New mutations of locus control region in Saudi sickle patients. Saudi J. Biol. Sci..

[17] Khan SA (2020). Socioeconomic status dependent medical complexities in children with sickle cell disease in Saudi Arabia. Saudi J. Biol. Sci..

[18] Erusalimsky JD (2009). Vascular endothelial senescence: from mechanisms to pathophysiology. J. Appl. Physiol..

[19] Erusalimsky JD, Skene C (2009). Mechanisms of endothelial senescence. Exp. Physiol..

[20] Platt OS (2000). Sickle cell anemia as an inflammatory disease. J. Clin. Invest..

[21] Wun T (2000). The role of inflammation and leukocytes in the pathogenesis of sickle cell disease. Hematology.

[22] Hebbel RP (2004). Special issue of microcirculation: examination of the vascular pathobiology of sickle cell anemia. Foreword. Microcirculation.

[23] Snyder AR, Zhou J, Deng Z, Lieberman PM (2009). Therapeutic doses of hydroxyurea cause telomere dysfunction and reduce TRF2 binding to telomeres. Cancer Biol. Ther..

[24] Drašar ER (2014). Leucocyte telomere length in patients with sickle cell disease. Br. J. Haematol..

[25] Colella MP (2017). Telomere length correlates with disease severity and inflammation in sickle cell disease. Rev. Bras. Hematol. Hemoter..

[26] Al-Attas OS (2010). Adiposity and insulin resistance correlate with telomere length in middle-aged Arabs: the influence of circulating adiponectin. Eur. J. Endocrinol..

[27] Al-Attas OS (2010). Telomere length in relation to insulin resistance, inflammation and obesity among Arab youth. Acta Paediatr..

[28] Al-Attas OS (2012). Circulating leukocyte telomere length is highly heritable among families of Arab descent. BMC Med. Genet..

[29] Cawthon RM (2002). Telomere measurement by quantitative PCR. Nucleic Acids Res..

[30] Quinn CT (2016). Minireview: clinical severity in sickle cell disease: the challenges of definition and prognostication. Exp. Biol. Med..

[31] Calado RT, Young NS (2009). Telomere diseases. N. Engl. J. Med..

[32] Fitzpatrick AL (2007). Leukocyte telomere length and cardiovascular disease in the cardiovascular health study. Am. J. Epidemiol..

[33] Wang J (2016). Association between telomere length and diabetes mellitus: a meta-analysis. J. Int. Med. Res..

[34] Wang L, Xiao H, Zhang X, Wang C, Huang H (2014). The role of telomeres and telomerase in hematologic malignancies and hematopoietic stem cell transplantation. J. Hematol. Oncol..

[35] Askree SH (2004). A genome-wide screen for *Saccharomyces cerevisiae* deletion mutants that affect telomere length. Proc. Natl Acad. Sci. USA.

[36] Uringa EJ, Youds JL, Lisaingo K, Lansdorp PM, Boulton SJ (2011). RTEL1: an essential helicase for telomere maintenance and the regulation of homologous recombination. Nucleic Acids Res..

[37] Gupta A (2013). Telomere length homeostasis responds to changes in intracellular dNTP pools. Genetics.

[38] Mainous AG (2013). Telomere length and elevated iron: the influence of phenotype and HFE genotype. Am. J. Hematol..

[39] Kozlitina J, Garcia CK (2012). Red blood cell size is inversely associated with leukocyte telomere length in a large multi-ethnic population. PLoS ONE.

[40] De Meyer T (2008). Lower red blood cell counts in middle-aged subjects with shorter peripheral blood leukocyte telomere length. Aging Cell.

[41] Lanaro C, Franco-Penteado CF, Conran N, Saad STO, Costa FF (2006). Anti-inflammatory effect of hydroxyurea therapy in sickle cell disease. Blood.

[42] Lanaro C (2009). Altered levels of cytokines and inflammatory mediators in plasma and leukocytes of sickle cell anemia patients and effects of hydroxyurea therapy. J. Leukoc. Biol..

[43] Liu Z, Zhang J, Yan J, Wang Y, Li Y (2014). Leucocyte telomere shortening in relation to newly diagnosed type 2 diabetic patients with depression. Oxid. Med. Cell Longev..

[44] Ma D, Zhu W, Hu S, Yu X, Yang Y (2013). Association between oxidative stress and telomere length in Type 1 and Type 2 diabetic patients. J. Endocrinol. Invest..

